# Compact Dual-Polarized Vivaldi Antenna with High Gain and High Polarization Purity for GPR Applications

**DOI:** 10.3390/s21020503

**Published:** 2021-01-12

**Authors:** Hai-Han Sun, Yee Hui Lee, Wenhao Luo, Lai Fern Ow, Mohamed Lokman Mohd Yusof, Abdulkadir C. Yucel

**Affiliations:** 1 School of Electrical and Electronic Engineering, Nanyang Technological University, 50 Nanyang Ave, Singapore 639798, Singapore; Haihan.Sun@ntu.edu.sg (H.-H.S.); wenhao.luo@ntu.edu.sg (W.L.); 2 Centre for Urban Greenery and Ecology, National Parks Board, 1 Cluny Rd, Singapore 259569, Singapore; genevieve_ow@nparks.gov.sg (L.F.O.); mohamed_lokman_mohd_yusof@nparks.gov.sg (M.L.M.Y.)

**Keywords:** dual polarization, ground-penetrating radar (GPR), high gain, low dispersion, polarization purity, ultrawide bandwidth, Vivaldi antenna

## Abstract

A compact ultra-wideband dual-polarized Vivaldi antenna is proposed for full polarimetric ground-penetrating radar (GPR) applications. A shared-aperture configuration comprising four Vivaldi elements for orthogonal polarizations is designed to reduce the low-end operating frequency and improve the port isolation with a compact antenna size. The directivity of the antenna is enhanced by the oblique position of the radiators and the implementation of a square loop reflector. Experimental results demonstrate that the antenna has very good impedance matching, port isolation, and dual-polarized radiation performance, with low dispersion characteristics across band of interest from 0.4 GHz to 3.0 GHz. GPR measurements with the designed antenna show that the antenna maintains good detection capability even for objects buried in a highly conductive soil.

## 1. Introduction

Ground-penetrating radar (GPR) has been widely used as a non-destructive method to detect and/or image subsurface objects and structures. The antenna is an essential component of the GPR system. Generally, GPR antennas operate at low frequencies of less than 3 GHz with an ultrawide bandwidth to guarantee high penetration depth and imaging resolution [1,2]. Many specifications are considered when designing the antenna to ensure GPR system performance under different scenarios. Directional and high-gain radiation characteristics are needed to increase the transmitted/received power and thus improve the detection and imaging capability [3]. A non-dispersive characteristic is essential to minimize the distortion of the waveform caused by the antenna. Underground objects, based on their orientation with respect to the polarization of electromagnetic (EM) waves, will depolarize the incident waves. Therefore, polarization diversity is useful for providing more information and improving detection capability [4]. A compact antenna size is preferred to obtain a lightweight and portable GPR system. For underground detection in tropical places (e.g., Singapore) where the soil moisture content is high and therefore the soil is relatively lossy, low operating frequencies with high gain performance are particularly important to increase the penetration depth of the EM waves into the ground.

So far, various ultra-wideband antennas have been experimentally studied for GPR applications, such as bowtie antennas [1,5], horn antennas [6], and tapered slot antennas (Vivaldi antennas) [3,7,8,9,10]. Among them, the Vivaldi antenna has been a popular choice due to its broad bandwidth, unidirectional radiation pattern, and ease of fabrication [7]. It has been used to construct GPR hybrid systems with high detection accuracy [8] and has also been employed to improve the imaging capability of buried objects by utilizing its high directivity characteristic [3].

Many approaches have been investigated to improve the radiation performance and structural characteristics of the Vivaldi antennas. The low-end operating frequency can be reduced and the radiation directivity at low band can be enhanced by incorporating defected ground structures [9], additional resonant cavities [11], and gradual corrugated edges [12] on the antenna aperture. In this way, the bandwidth of the antenna is broadened without increasing the aperture size. The connected array and tightly coupled array configurations have also been utilized to reduce the lowest operating frequency of Vivaldi antennas [13,14,15]. By directly connecting the radiating fins of the Vivaldi elements, optimizing the number of elements, and adopting suitable feed networks, the low cut-off frequency can be greatly reduced. Directivity at high frequencies can be enhanced by placing directors in front of the antenna aperture. The adopted directors can be of different shapes and materials, and include triangular metal directors [12], planar strip directors [16], directors formed by high-permittivity materials [17], and anisotropic zero-index metamaterials [18]. In [19], a double slot structure was presented to obtain a uniform field distribution at the end of the antenna for enhancing the directivity. However, most of the proposed antennas are single-polarized and their dispersion characteristics have not been investigated.

Dual-polarized Vivaldi antennas are generally realized by orthogonally inserting two Vivaldi elements into each other, forming a cross shape [20,21,22,23,24]. The feeding lines of the elements need to be slightly shifted to avoid their intersection and to improve port isolation. Microstrip-to-slot balun [22] and two-stage quarter-wave balun [23] have been used to achieve a broad bandwidth. An ultra-lightweight dual-polarized antenna design was developed using glass fiber-reinforced epoxy with copper layers [24]. Most of the dual-polarized antennas presented have a wide operating bandwidth with a moderate port isolation level of around 25 dB, cross-polarization discrimination of less than 20 dB, and a moderate averaged gain of about 5.5 dBi. The electrical sizes of these antennas are often relatively large, which limits their utilization in portable GPR systems.

To improve the port isolation and enhance the gain while maintaining a compact antenna size, we present a shared-aperture dual-polarized Vivaldi antenna. The novel aspects of the proposed antenna are three-fold: (1) a shared-aperture configuration comprising four Vivaldi elements is introduced to achieve dual polarization with enhanced bandwidth and improved port isolation while maintaining a very compact antenna size; (2) a unique oblique arrangement of Vivaldi elements in the array is presented to improve the radiation performance; and (3) a loop reflector is specially designed to enhance the low-frequency gain while maintaining low dispersion characteristics of the antenna. After incorporating all these novel ideas and implementations into the design, a significant enhancement of the antenna performance characteristics was achieved. Experimental results showed that the antenna has a broad bandwidth of 164%, high port isolation above 40 dB, enhanced gain of 4–12 dBi, high polarization purity, and low dispersion with a very compact antenna size of 0.28 × 0.28 × 0.20 λ^3^. The performance characteristics make the antenna an excellent candidate for a compact, fully polarimetric GPR system. GPR experiments using the fabricated antennas have been carried out in a true soil environment in Singapore. The underground objects were successfully detected with good accuracy and resolution.

## 2. Antenna Design and Operating Mechanism

### 2.1. Antenna Configuration

**Figure 1:**
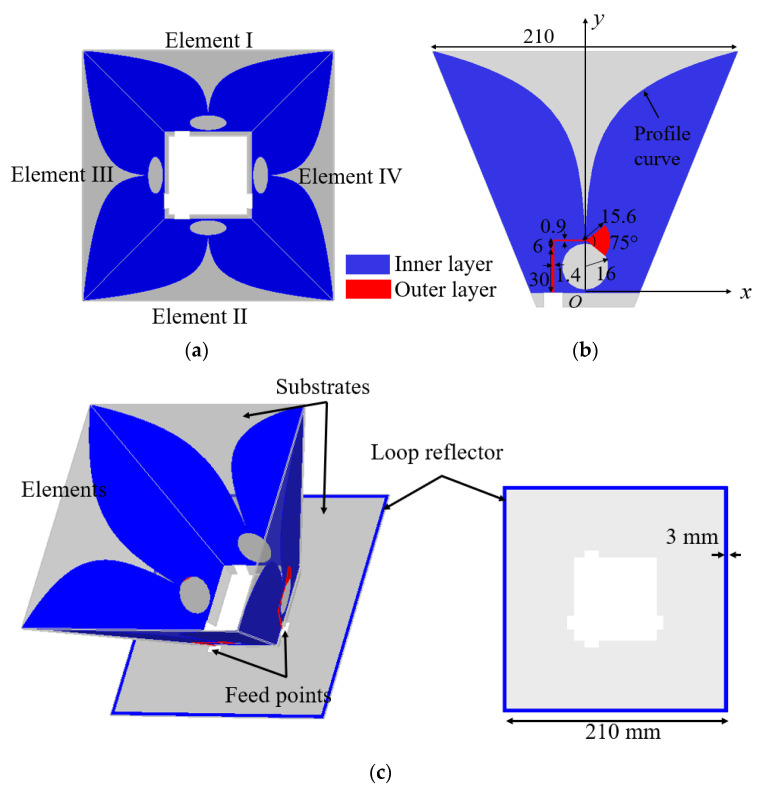
Configuration of the proposed dual-polarized Vivaldi antenna: (**a**) Top view and (**c**) perspective view. (**b**) Configuration of each Vivaldi element. Dimensions are in mm unless specified.

The configuration of the dual-polarized Vivaldi antenna is shown in Figure 1. Four obliquely positioned Vivaldi antenna elements are connected in a horn shape. Each Vivaldi element is fed by an optimized microstrip-slot balun. Two parallel elements are excited simultaneously with equal magnitude and phase for one polarization, so two orthogonal polarizations can be realized by exciting elements I and II, and elements III and IV, respectively. A thin square metallic loop is placed at the bottom of the antenna and acts as a reflector. Details of a single Vivaldi element with optimized dimensions are shown in Figure 1b. The exponential profile curves employed in this design (in mm) are calculated by:

(1) $$x=\pm \left(0.27e^{0.036y}-0.78\right).$$

The endpoints of the profile curves are *x* = ±0.2 mm, *y* = 36 mm, and *x* = ±105 mm, *y* = 166 mm. The substrates used for the Vivaldi elements and the reflector are F4BTM-2 and F4B, with relative permittivity of 4.4 and 2.2, respectively. Both substrates have a loss tangent of 0.0025 and a thickness of 1.0 mm. The antenna has a size of 210 × 210 × 152 mm^3^. The antenna was designed to cover the operating band from 0.4 GHz to 3.0 GHz to guarantee high penetration depth and resolution when used for GPR applications. CST Studio Suite 2019 was used to design and simulate the antenna in this work.

The proposed design is a combination of a number of working principles: the implementation of a novel shared-aperture configuration, the oblique arrangement of Vivaldi elements, and the use of a square loop reflector.

### 2.2. Working Principle Analysis

#### 2.2.1. The Shared-Aperture Configuration

**Figure 2:**
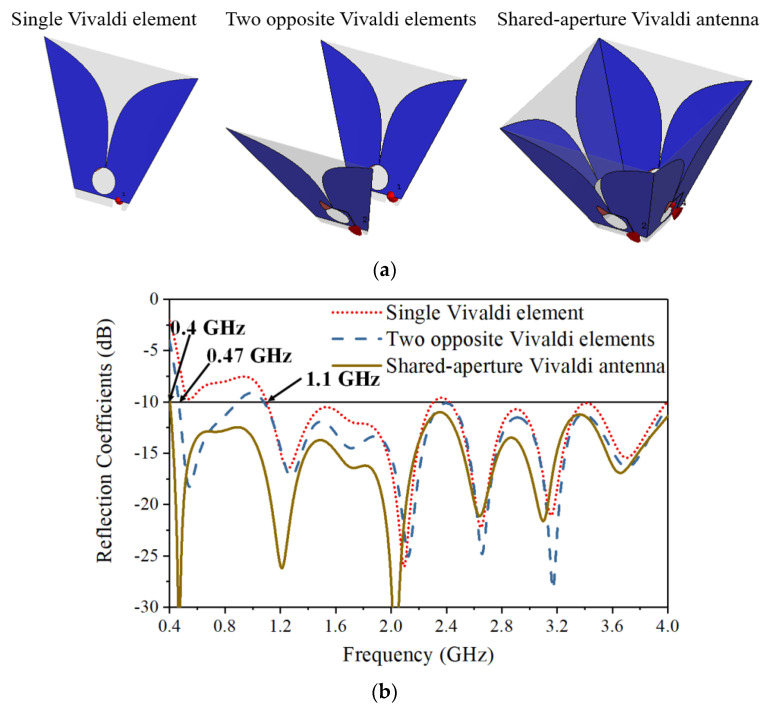

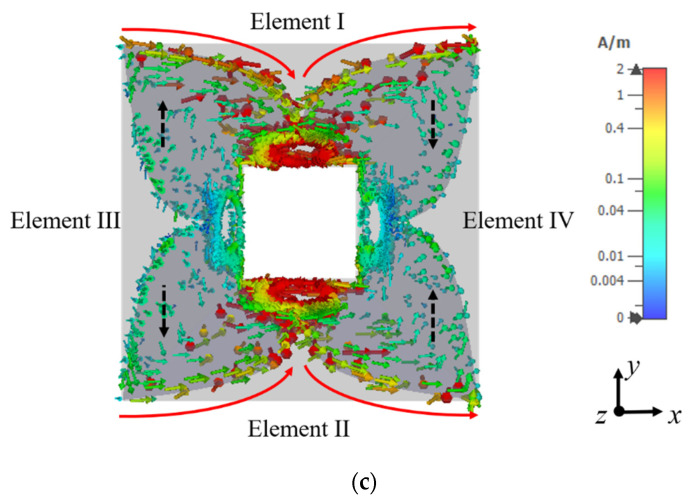
(**a**) Configuration of a single Vivaldi element, two opposite Vivaldi elements, and the shared-aperture Vivaldi antenna. (**b**) Comparison of reflection coefficients in the three cases. (**c**) Current distribution on the shared-aperture Vivaldi antenna at 0.5 GHz.

The shared-aperture configuration has significant advantages in broadening the operating frequency and improving isolation between orthogonal ports with a compact antenna size. The design procedure starts with a single Vivaldi element, followed by the addition of an opposite Vivaldi element and the combination of their radiations for one polarization, and finally the introduction of another two orthogonal elements for the orthogonal polarization and connection of the four elements together, as illustrated in Figure 2a. The reflection coefficients in the three cases are shown in Figure 2b. Adding an opposite element introduces a strong mutual coupling between the two elements for the same polarization, which helps reduce the lowest operating frequency of the structure from 1.1 GHz to 0.47 GHz. The addition of the other two orthogonal connecting elements further reduces the low-end operating frequency to 0.4 GHz and improves the reflection coefficient by around 3 dB for frequencies below 2 GHz.

The current distribution at 0.5 GHz when elements I and II are excited for horizontal polarization is shown in Figure 2c. Most of the currents are distributed on the driven elements with a small amount of current flowing on the connected elements III and IV. Some current paths at low frequencies extend to the connected elements, which helps to slightly reduce the low-end operating frequency and improve the impedance matching at low frequencies. In addition, the currents on the two radiating fins of connecting elements III and IV are equal in magnitude but out of phase in the *y*-direction, thus providing a negligible contribution to the cross-polarized radiation. Theoretically, the resulting voltage across the feeding points of elements III and IV is zero. Therefore, a very high isolation level between the orthogonal ports can be achieved.

#### 2.2.2. The Oblique Arrangement of Vivaldi Elements

**Figure 3:**
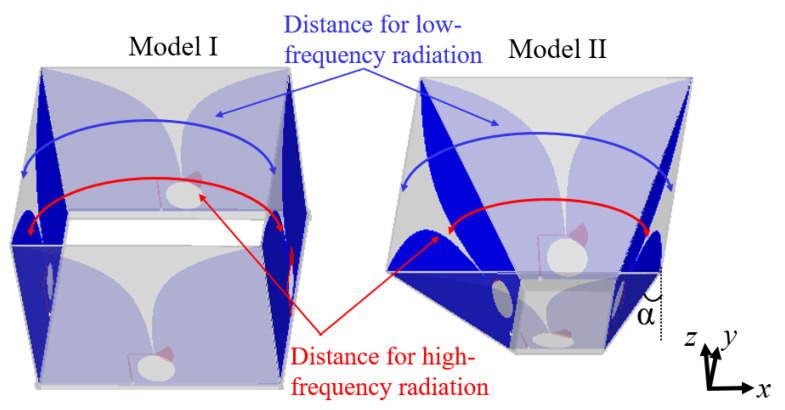
Configuration of Model I (vertically arranged Vivaldi elements) and Model II (obliquely arranged Vivaldi elements).

As the two Vivaldi elements with the same polarization are placed facing each other and are excited simultaneously, an array factor is included for the combined radiation pattern. A fixed distance between two Vivaldi elements that is suitable for low frequencies would result in a high sidelobe level at high frequencies and a low directivity. One unique feature of the Vivaldi element is that the EM waves radiate from a wide area of the open slot at low frequencies and from a narrow area of the slot at high frequencies. Utilizing this feature, we arranged the elements at an oblique angle of α to reduce the distance between radiating surfaces at high frequencies while having minimal impact on the larger distance required at low frequencies, as illustrated in Model II in Figure 3. In this way, with a given angle α, the directivity at certain high frequencies can be improved with a lower sidelobe. Here we need to note that the tapered or curved surfaces can be used to optimize the spacing between the Vivaldi elements to provide better directivity enhancement over a broad bandwidth. However, for ease of fabrication, a straight surface is considered.

**Figure 4:**
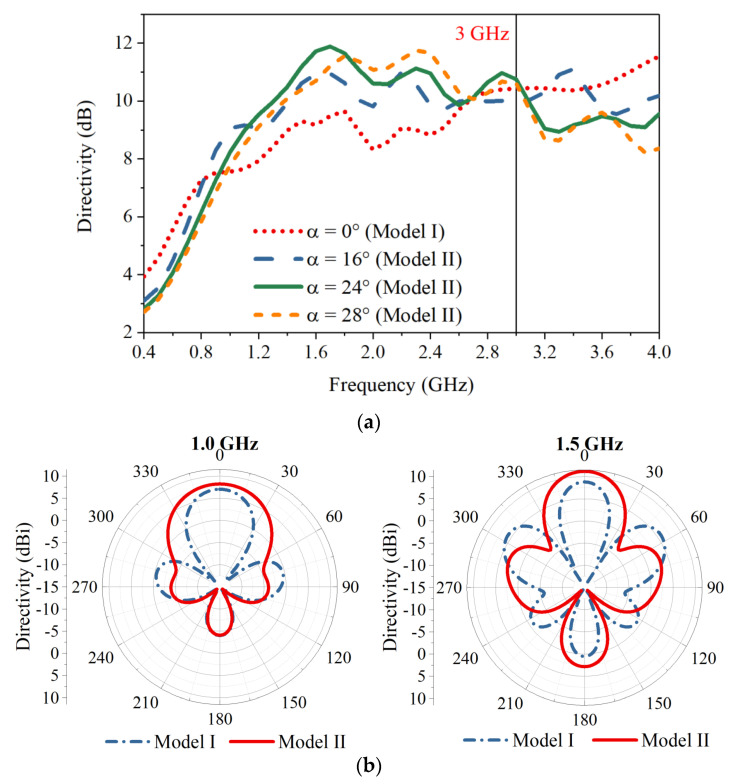
(**a**) Comparison of the antenna directivity of Model I (vertically arranged Vivaldi elements) and Model II (obliquely arranged Vivaldi elements) with different values of α. (**b**) The H-plane radiation patterns at 1.0 GHz and 1.5 GHz of Model I and Model II (α = 24°).

The antenna directivity of the vertically arranged configuration (Model I, where α = 0°) and the obliquely arranged configuration (Model II, with different oblique angle α) is compared in Figure 4a. Across the band of interest for the GPR application (0.4–3.0 GHz), the directivity of Model I increases with frequency and has a peak value of 10 dB at 3.0 GHz. Compared with the directivity of Model I, by obliquely arranging the Vivaldi elements, the directivity of Model II is increased noticeably at frequencies above 0.8 GHz. A larger α in Model II enhances the directivity at a higher frequency band, but slightly reduces the directivity at low frequencies. In order to maintain a high average directivity across the band of interest, α in the design is chosen to be 24°. This leads to a maximum directivity increment of 2.7 dB with a peak value of 11.9 dB at 1.6 GHz. The performance at lower frequency is slightly compromised with a decrease in the directivity of less than 1 dB. For frequencies higher than 3.0 GHz, the directivity of Model II is lower than that of Model I due to the limited influence of the oblique arrangement in the distance between the radiating areas at these frequencies. The distance is still very large in terms of wavelength, which results in high sidelobe levels and reduced directivity. Nevertheless, the maximum directivity of Model II maintains an acceptable level of 9 dB for frequencies higher than 3.0 GHz.

The comparison of H-plane (*xz*-plane) radiation patterns at 1.0 GHz and 1.5 GHz of the two models I and II with α = 24° is shown in Figure 4b. The sidelobe levels are reduced by 4.8 dB and 5.3 dB at the two respective frequencies with the proposed oblique arrangement, demonstrating the effectiveness of the oblique arrangement in reducing the sidelobe levels and enhancing the directivity at these frequencies.

#### 2.2.3. The Square Loop Reflector

**Figure 5:**
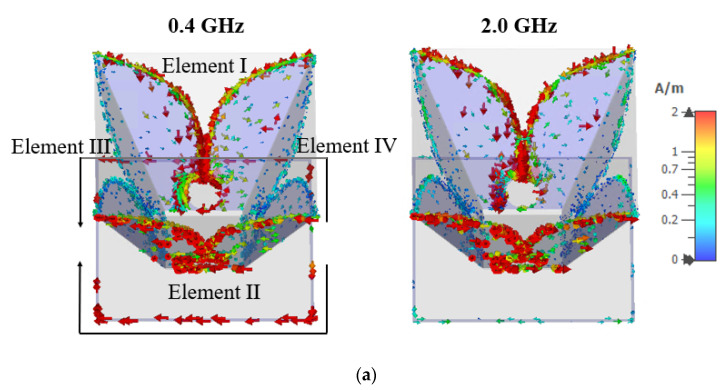

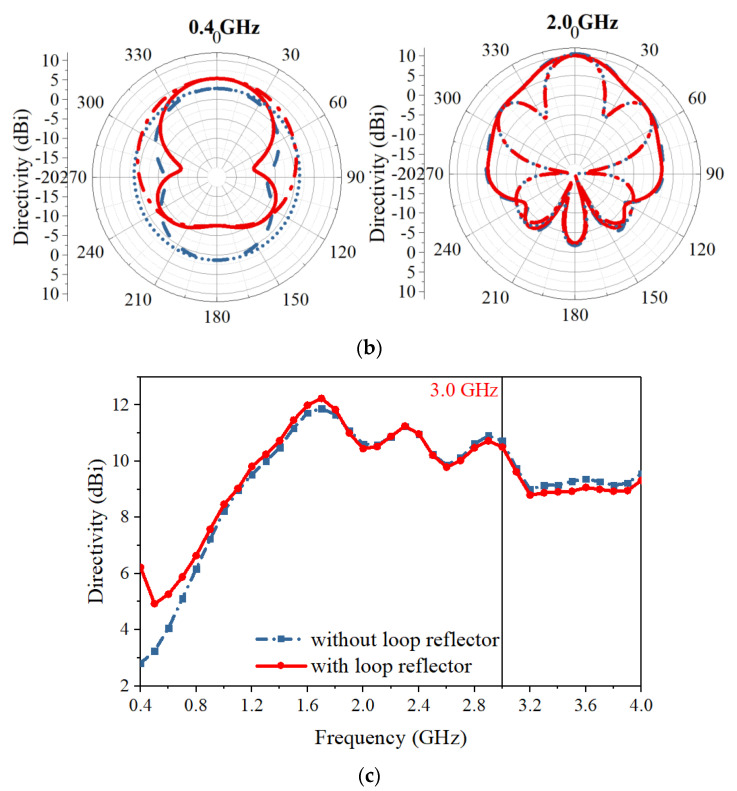
(**a**) Current distribution on the antenna with the loop reflector at 0.4 GHz and 2.0 GHz; (**b**) comparison of radiation patterns with and without the square loop reflector at 0.4 GHz and 2.0 GHz; and (**c**) antenna directivity from 0.4 GHz to 4.0 GHz.

It can be observed in Figure 4a that the directivity at low frequencies is much lower than that at high frequencies. Planar or cavity reflectors can be effective options to improve the directivity at lower frequencies [25,26], but reflections from a large metallic reflector will give rise to unwanted ringing effect and result in waveform distortion, which is undesirable for GPR applications [27]. In this work, a thin square metallic loop is implemented as a reflector to improve the directivity at low frequencies while minimizing its impact on the antenna dispersion. The configuration of the loop reflector is shown in Figure 1c. The circumference of the metallic loop is 840 mm, which is slightly larger than a wavelength at 0.4 GHz. The current distributions on the antenna at 0.4 GHz and 2.0 GHz when elements I and II are excited are illustrated in Figure 5a. At 0.4 GHz, a large amount of current is induced on the thin square loop, forming two current paths. The length of each current path is approximately λ/2 at 0.4 GHz. The radiation from this current is combined with the radiation from the elements I and II, leading to an enhancement in directivity by 3.4 dB, as shown in Figure 5b. For current distribution at higher frequencies such as at 2.0 GHz, small currents are observed on the square loop, so the loop reflector has minimal impact on the radiations at high frequencies, as shown in Figure 5b. The directivity of the antenna with and without the loop reflector is compared in Figure 5c. The existence of the reflector increases the directivity from 0.4 GHz to 0.9 GHz, with a maximum increment of 3.4 dB at 0.4 GHz.

The final antenna structure combining the shared-aperture configuration, the oblique arrangement of Vivaldi elements, and the loop reflector has a resultant directivity that varies from 5.0 dB to 12.2 dB within the operating frequency band.

## 3. Antenna Performance

### 3.1. Antenna Radiation Characteristics

**Figure 6:**
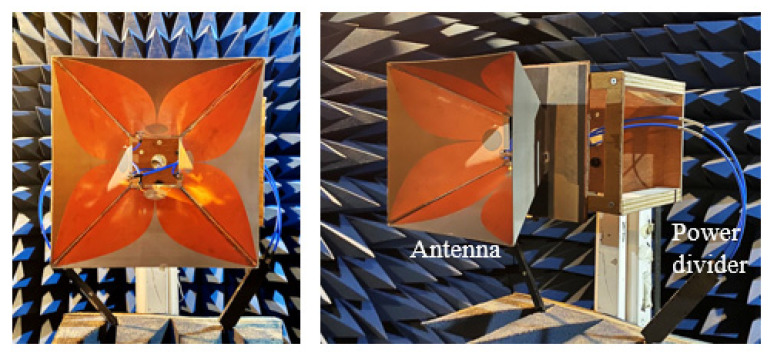
Front and perspective views of the fabricated prototype.

The antenna prototype was fabricated as shown in Figure 6. Four Vivaldi elements were soldered together via their edges, forming a horn shape. Silicone adhesive was used to assemble the reflector and seal all the gaps in the antenna to improve the structure robustness [23]. Two groups of orthogonally polarized elements were fed by commercial power dividers (PWD-2W-0.5G-6G-10W-Sf) for simultaneous excitation.

**Figure 7:**
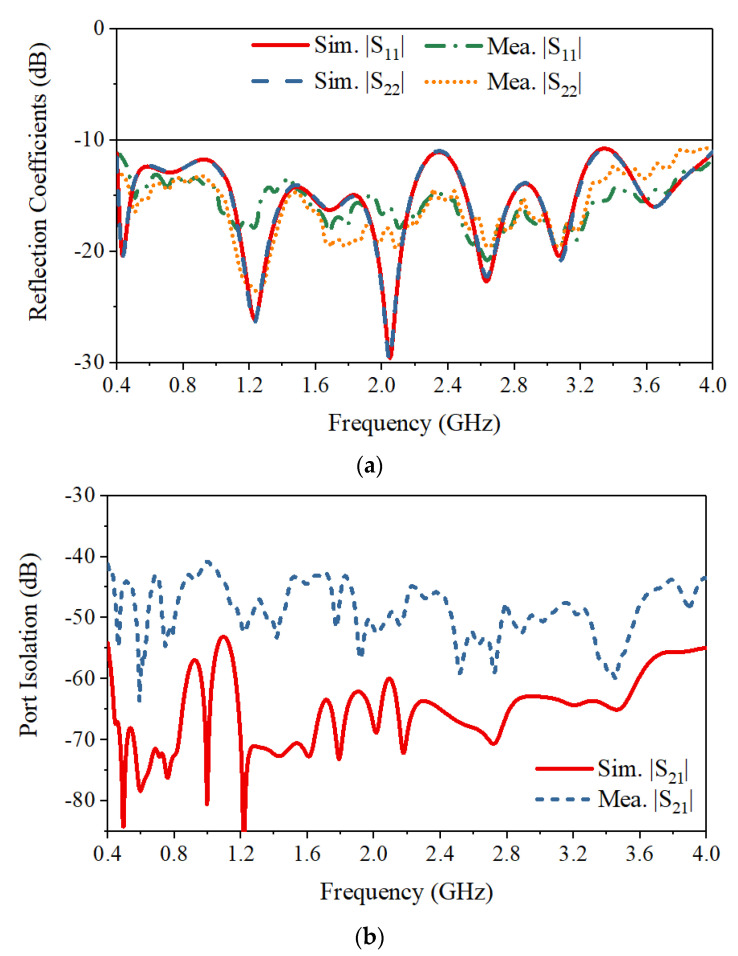
Simulated and measured (**a**) reflection coefficients, and (**b**) port isolation.

**Figure 8:**
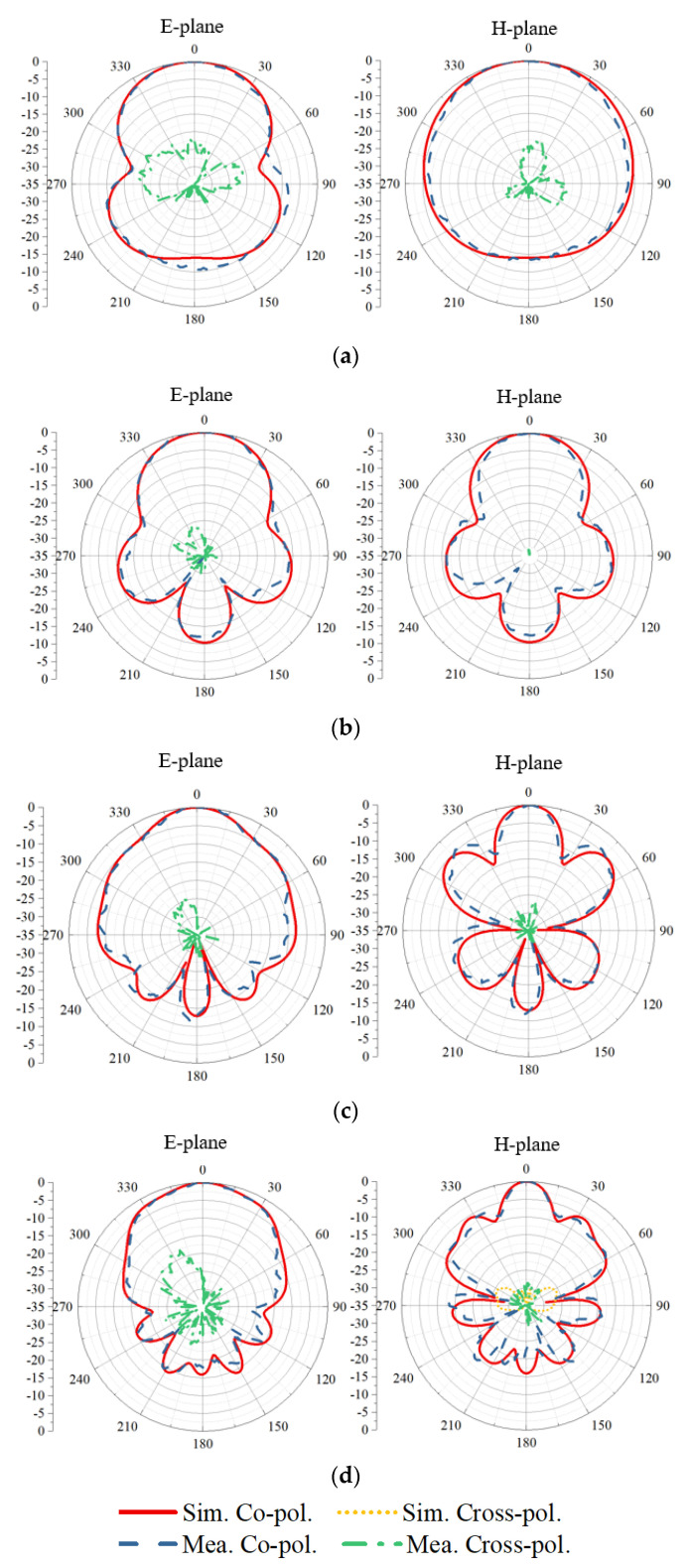
Normalized radiation patterns in E- and H-planes at (**a**) 0.4 GHz, (**b**) 1.2 GHz, (**c**) 2.0 GHz, and (**d**) 2.8 GHz.

**Figure 9:**
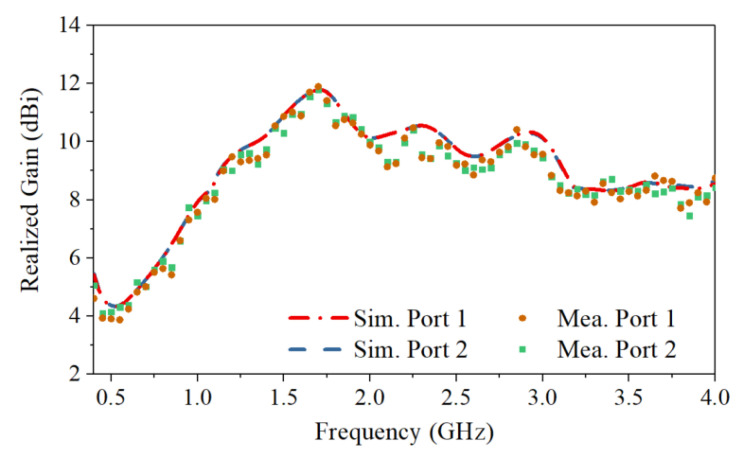
Simulated and measured maximum realized gain of the antenna.

**Figure 10:**
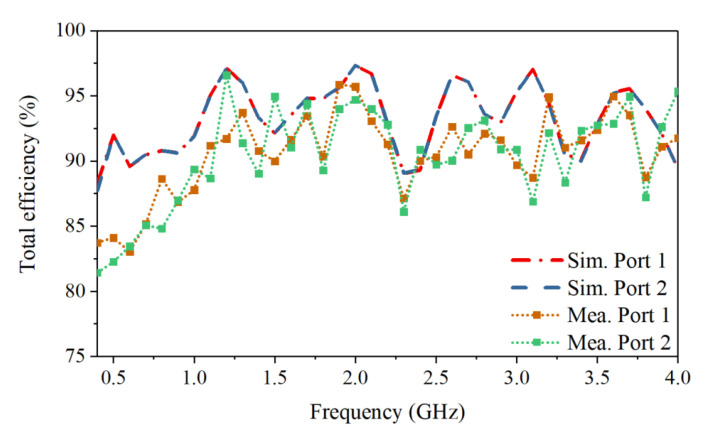
Simulated and measured total efficiency of the antenna.

The S-parameters obtained by simulation and measurement are shown in Figure 7. The measured reflection coefficients of the two ports are in good agreement with the simulated ones. The measured operating frequency is from 0.4 GHz to 4.0 GHz with reflection coefficients lower than −10 dB. Across the band, the measured port isolation is more than 40 dB, as shown in Figure 7b. The isolation is calculated using the S4P parameters of the antenna with an ideal power divider to eliminate the impact of the imbalance of the real divider. The discrepancy between the simulated and measured port isolations can be caused by the fabrication tolerance and imperfect soldering. The normalized simulated and measured radiation patterns for horizontal polarization in both the E- and H-planes are shown in Figure 8. The results for the vertical polarization are identical to those for the horizontal polarization due to the symmetrical configuration of the antenna. As shown in Figure 8, the measured radiation patterns are in good agreement with the simulated ones, and both show directive radiation with cross-polarization discrimination (XPD) higher than 23 dB at boresight. The XPD is calculated by $20\log_{10}\left(E_{co-pol.}\left(\theta \right)/E_{cross-pol.}\left(\theta \right)\right)$. The antenna has a measured gain that varies from 4 dBi to 12 dBi, as presented in Figure 9. The total efficiency of the antenna is more than 82% across the operating band, as shown in Figure 10.

Table 1 compares the radiation performance of the proposed antenna (Ant.) with other reported ultra-wideband dual-polarized Vivaldi antennas. Clearly, the proposed antenna achieves higher port isolation, higher polarization purity, and higher gain with a very compact aperture size compared to other ultra-wideband dual-polarized Vivaldi antennas.

### 3.2. Antenna Dispersion

The ringing effect of an antenna can distort the transmitted and received signals and disguise the target signals with noisy reflections from the environment. Low antenna dispersion is required to guarantee the detection accuracy of the GPR system. The dispersion can be investigated in both the time and frequency domains.

**Figure 11:**
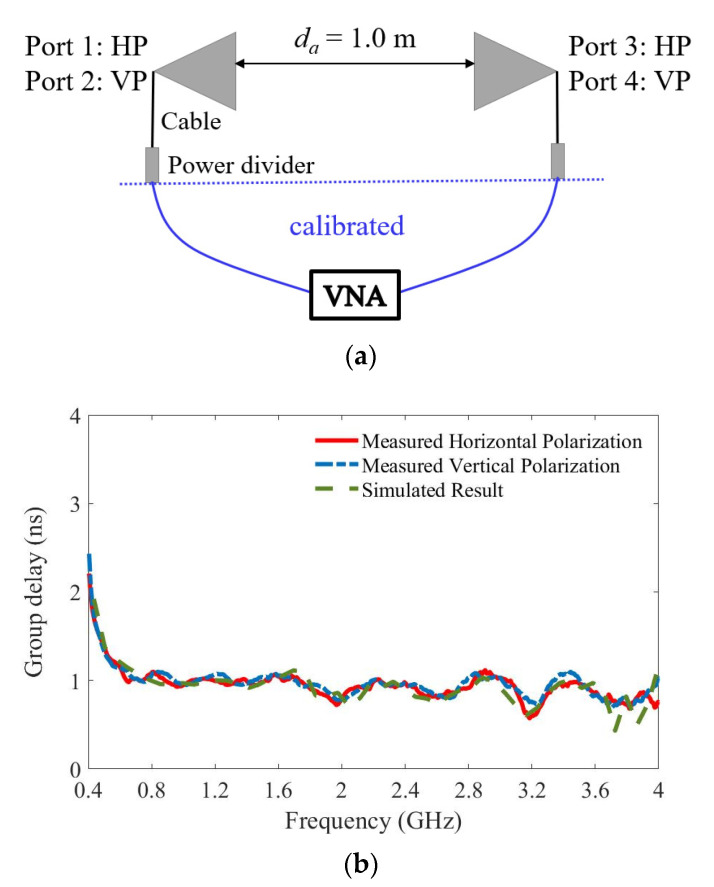
(**a**) Illustration of the group delay measurement. (**b**) The calculated group delay using the simulated and measured results of the proposed dual-polarized Vivaldi antenna. VNA: Vector Network Analyzer.

In the frequency domain, the dispersion is evaluated by the group delay, which represents the delay of the amplitude envelopes of a signal at various frequencies through the antenna. A constant group delay is required to guarantee minimum distortion of a signal. The group delay of the proposed Vivaldi antenna is measured by placing two antennas face to face at a distance of 1.0 m, as illustrated in Figure 11a. As the antenna has two polarizations, the group delays in both cases are examined. *S*_31_ and *S*_42_ are measured and their phase components are extracted as

(2) $$\Phi_{S31}\left(f\right)=2\times \Phi_{a}\left(f\right)+\Phi_{pc}\left(f\right)+\Phi_{d}\left(f\right)$$

where $\Phi_{a}$, $\Phi_{pc}$, and $\Phi_{d}$ represent the phase delay due to the transmission of the signal through the antenna, the power dividers and cables, and the distance $d_{a}$ between two antennas in free space, respectively. Here, $\Phi_{pc}$ is measured by connecting the cables directly at their ends, and $\Phi_{d}$ is calculated via $2\pi fd_{a}/c$, where *c* is the speed of light. All the phases must be unwrapped correctly in the calculation. The group delay of the antenna is expressed as

(3) $$Group\;delay=-\frac{1}{2\pi }\cdot \frac{d}{df}\Phi_{a}\left(f\right)$$

Figure 11b plots the calculated group delay using the simulated and measured results. All the curves show a constant group delay with variations of less than 1 ns across most of the band.

In the time domain, the ringing effect can be quantified by the transient response of the antenna. The antenna’s transient response is defined by modeling the antenna as a linear time-invariant transmission system with excitation voltage and radiated field strength as input and output parameters, respectively [28]. The duration of the ringing in the transient response should be less than a few multiples of the duration of its main pulse for an impulse ultra-wideband antenna.

**Figure 12:**
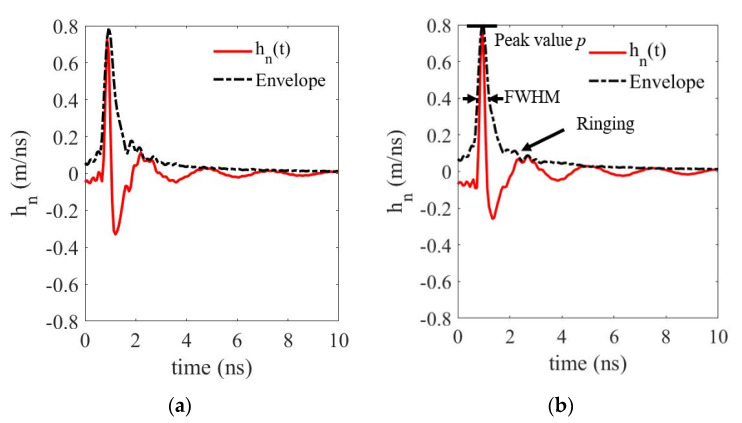
(**a**) Simulated and (**b**) measured impulse response of the proposed dual-polarized Vivaldi antenna. FWHM: full width at half maximum of the magnitude.

With two identical antennas for transmission and reception, the antenna’s co-polarized transient response $h_{n,copol}^{+}\left(k\Delta t\right)$ can be derived from its transfer function $H_{n,copol}^{+}\left(f\right)$, and they are calculated using [29]

(4) $$h_{n,copol}^{+}\left(k\Delta t\right)=\frac{1}{N\Delta t}\overset{N-1}{\underset{n=0}{\sum }}H_{n,copol}^{+}\left(f\right)e^{j\left(\frac{2\pi }{N}\right)kn}$$

(5) $$H_{n,copol}^{+}\left(f\right)=\{\begin{array}{ll} 2H_{n,copol}\left(f\right) & for\;f>0\\ 0 & for\;f\leq 0 \end{array}$$

(6) $$H_{n,copol}\left(f\right)=\sqrt{\frac{d_{a}c}{jf}S_{31}\left(f\right)e^{\frac{j2\pi fd_{a}}{c}}}$$

where *S*_31_ (or *S*_42_) is the measured *S* parameters for horizontal (or vertical) polarization (HP/VP). *N* is the total number of points in the inverse discrete Fourier transform, and *k* and *n* are the indices of the sampled points in the time and frequency domains, respectively. Zero padding for 0–400 MHz and 4.0–15 GHz is used for a fine interpolation of the antenna’s transient response. The results for the horizontal and vertical polarizations are the same due to the symmetric configuration of the antenna. The simulated and measured transient response in the main beam direction for horizontal polarization is plotted in Figure 12. The measured results resemble the simulated one. The envelopes show a sharp peak and a fast decay of the ringing.

The parameters defined in [29] to evaluate the antenna’s transient response are the peak value *p*, the full width at half maximum of the magnitude (FWHM), and the duration of the ringing $\tau_{r}$ for the magnitude of the envelope falling from p to a certain lower bound r·p. For the measured transient response of the proposed antenna in the main beam direction, the parameters are p = 0.8 m/ns, FWHM = 0.46 ns, and $\tau_{r}$ = 0.60 ns for *r* = 0.22. The duration of the ringing is only 1.3 times the FWHM, indicating the antenna has low dispersion and short ringing characteristics [28].

## 4. GPR Experimental Validation

**Figure 13:**
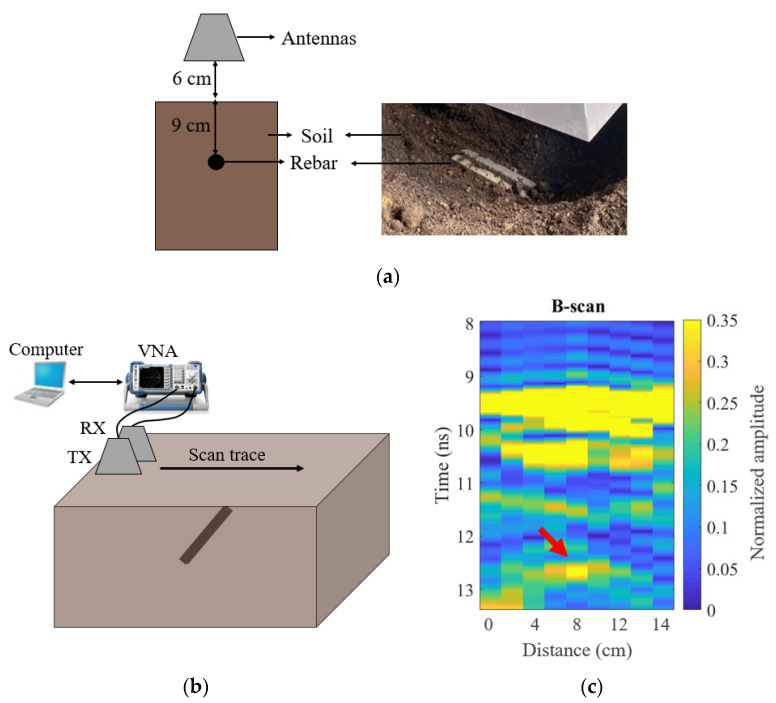
(**a**) Demonstration of the testbed setup for the GPR measurement with the proposed Vivaldi antenna. (**b**) Illustration of the GPR system and the experiment. (**c**) Normalized B-scan results of the GPR measurement. TX and RX are the transmitting and receiving Vivaldi antennas, respectively.

Experiments were carried out to evaluate the performance of the proposed antenna in a real GPR setting, as shown in Figure 13a,b. The testbed had a size of 1200 × 800 × 400 mm^3^ and was filled with soil. The transmitting and receiving Vivaldi antennas (TX and RX) were separated with a side-to-side distance of 10 cm and were mounted 6 cm above the soil. The transmission coefficient S_21_ between the two antennas was measured using a Vector Network Analyzer (VNA). Although the antenna could perform well up to 4.0 GHz, high-frequency signals mainly contained environmental noise rather than signals from underground objects due to the attenuation of the soil. Therefore, in the GPR experiment, the operating frequency of the antennas was selected from 0.4 GHz to 3.0 GHz. A metal rebar with a size of 200 × 40 × 40 mm^3^ was buried at 9 cm in the soil. The soil was of the approved soil mix (ASM) for general landscaping in Singapore [30]. The dielectric properties of the soil were measured using the Agilent 85070D Dielectric Probe. The averaged relative permittivity was around $\varepsilon_{r}=28-j2.5$ and the loss tangent was calculated by $\tan\delta =\varepsilon^{\prime \prime }/\varepsilon^{\prime }=0.09$. The conductivity of the soil was relatively high due to the high moisture content of soil in Singapore; it is challenging to detect the objects within this soil environment.

In the experiment, the antennas were moved along a scan trace above the buried target with a step of 2 cm, as shown in Figure 13b. At each position *m*, we could acquire a radar trace as a function of two-way travel time and signal amplitude by taking the inverse Fourier transform of the transmission coefficient S_21_ between TX and RX. This radar trace is denominated the A-scan [31,32]. The amplitude of signal at time sample *n* in radar trace *m* can be expressed as $A_{m}\left(n\right)$. Multiple consecutive A-scans along the scan trace can be further combined into a radargram called B-scan where the horizontal axis is the antennas’ location and the vertical axis is the two-way travel time of the signal [31,32]. The signal amplitude matrix of B-scan at time sample *n* in radar trace *m* can be expressed as B(m, n). The background removal operation was carried out to reduce clutter by subtracting from each A-scan the average value of the amplitude at a time sample assessed over all the A-scans [32]. The procedure is expressed as

(7) $$A_{m}^{\prime }\left(n\right)=A_{m}\left(n\right)-\frac{1}{M}\overset{M}{\underset{m=1}{\sum }}A_{m}\left(n\right)$$

where $A_{m}^{\prime }\left(n\right)$ and $A_{m}\left(n\right)$ are the processed and raw radar traces, respectively, and *M* is the total number of radar traces.

Figure 13c shows the measured B-scan after applying background removal. The rebar was visible as a hyperbolic shape starting at 12.5 ns. The reflected signal from the soil surface was located at 9.2 ns. The calculated depth based on the measured time delay difference between the reflected signals from the soil surface was 9.3 cm. The speed of EM waves in soil used in the calculation was $c/\sqrt{\left|\varepsilon_{r}\right|}$. The slight difference between the calculated depths and the real depths could be caused by the heterogeneous soil media with inconsistent permittivity. The noise in the images came from the reflections from the rough soil surface, embedded stones, and wet clods in the true soil environment [33], as well as reflections from the surrounding environment. Signal processing techniques are being investigated to improve the image quality further.

## 5. Conclusions

In this paper, a dual-polarized Vivaldi antenna was proposed for GPR applications. In the design, a shared-aperture configuration with four Vivaldi elements for orthogonal polarizations was used to broaden the bandwidth with a compact aperture size. Oblique arrangement of the Vivaldi elements and a square loop reflector were implemented to improve the directivity at the high and low frequencies, respectively. The antenna had a wide impedance matching bandwidth from 0.4 GHz to 4.0 GHz and high port isolation of more than 40 dB with a compact aperture size of 0.28 × 0.28 × 0.20 λ^3^ at 0.4 GHz. Both the radiation performance and the antenna dispersion characteristics were investigated for the proposed antenna. The antenna preserved good directivity, with high polarization purity and high gain. The group delay and the transient response results showed that the antenna had a low dispersion and a fast decay of the ringing, which are important factors for a GPR antenna. The GPR measurement of a buried rebar was presented to demonstrate the applicability of the proposed antenna to the GPR systems. The ultra-wideband dual-polarized radiation performance with high gain, excellent polarization purity, and low dispersion characteristics, together with the compact aperture size, are characteristics that make the proposed antenna a good candidate for full polarimetric GPR applications.

## Figures and Tables

**Table 1:** Comparison of antenna performance.

Ref.	Freq. (GHz)/fBW	Isolation (dB)	XPD (dB)	Gain (dBi)	Size (λ^3^) ^1^
[21]	3.1–10.6/109%	20	15	Max. 10	0.36 × 0.36 × 0.55
[22]	0.68–7.3/166%	30	19	3.8–11.2	0.36 × 0.36 × 0.56
[23]	0.56–7.7/173%	28	17.2	1.2–9.2	0.24 × 0.24 × 0.35
[24]	0.5–3.0/143%	15	NG	1.0–8.6	0.33 × 0.33 × 0.30
**Ant.**	**0.4–4.0/164%**	**40**	**23**	**4.0–12.0**	**0.28 × 0.28 × 0.20**

^1^ λ is the wavelength at the lowest operating frequency of the antenna. XPD: cross-polarization discrimination.

## Data Availability

Data sharing not applicable.
